# The burden of disease attributable to high body mass index across Arab countries: an analysis of data from the global burden of disease study 2021

**DOI:** 10.1186/s12889-025-25620-w

**Published:** 2025-11-26

**Authors:** Holly Exton-Smith, Ishani Sharma, Zeenah Atwan, Fakhria Al Rashdi, Hamed Al-Qanubi, Salman Rawaf, Celine Tabche

**Affiliations:** 1 School of Public Health, Imperial College London, London, UK; 2 School of Medicine, Imperial College London, London, UK; 3 WHO Collaborating Centre, School of Public Health, Imperial College London, London, UK; 4 Central Laboratory, College of Medicine, University of Basrah, Basrah, Iraq

**Keywords:** Obesity, Body mass index, Cardiovascular diseases, Diabetes, Kidney diseases, Global burden of disease study, Arab countries

## Abstract

**Background:**

Overweight and obesity are major risk factors for numerous communicable and non-communicable health conditions. Some of those diseases which can be prevented are cardiovascular diseases, diabetes, and chronic kidney disease. In Arab countries, prevalence of overweight and obesity is double what it is globally, making obesity a top public health concern. This study seeks to investigate the regional burden of high BMI-attributable disease and compare it with global data, to shed light on this public health issue and inform future policies.

**Methods:**

Data from the Global Burden of Disease (GBD) Study 2021, including global and Arab countries statistics, were systematically extracted and analysed. A high body mass index (BMI) was defined as a value of 25 kg/m² or greater. Trends for nine major causes of high BMI-attributable deaths, disability-adjusted life years (DALYs), and years of life lost from mortality (YLLs) were analysed by age and sex, from 1990 to 2021.

**Results:**

In Arab countries, the top causes of high BMI-attributable age-standardised deaths, DALYs, and YLLs in 2021 were cardiovascular diseases, diabetes and kidney diseases, and neoplasms (replacing chronic respiratory diseases in 1990). There was a notable increase in the disease burden attributable to high BMI between 1990 and 2021, with a consistently higher burden compared to global data. Mortality caused by cardiovascular diseases and diabetes and kidney diseases in Arab countries was higher in females than males, compared to global mortality data.

**Conclusions:**

The considerable disease burden attributable to high BMI in Arab countries highlights the necessity for integrated, population-level interventions aimed at holistically preventing high BMI in this region. Longitudinal and qualitative research on perceptions and drivers of trends in high BMI within Arab countries are necessary to gain context and add to the evidence base, allowing for the development of suitable and effective interventions.

**Supplementary Information:**

The online version contains supplementary material available at 10.1186/s12889-025-25620-w.

## Background

Body mass index (BMI) is a core component of health risk calculators, including QRisk3, which predicts the ten-year risk of cardiovascular disease (CVD) events, such as myocardial infarction (MI) and stroke [1]. Although BMI is an imperfect indicator of health and CVD risk, primarily as it fails to account for the cardioprotective effect of muscular tissue mass, it remains a common reference marker for the general population [2, 3]. In public health contexts, BMI thresholds are used to categorise weight status: overweight is defined as a BMI greater than or equal to 25 kg/m² in adults, while obesity is defined as a BMI greater than or equal to 30 kg/m² [3]. Obesity is a multifactorial condition attributed to an obesogenic environment (including socioeconomic status, food environment and policy factors), genetic and epigenetic factors, and recently, climate change and increased atmospheric temperatures [3, 4].

Obesity is a major risk factor for a wide range of physical and mental health conditions. It is associated with a fivefold increased risk of type two diabetes and a 2.5-times risk of hypertension; there is a strong correlation between obesity and some cancers, such as colon cancer [5]. Beyond these, obesity is also strongly associated with conditions such as non-alcoholic fatty liver disease (NAFLD), non-alcoholic steatohepatitis (NASH), CVD, asthma, and obstructive sleep apnoea (OSA) [6]. Furthermore, individuals with obesity were shown to have higher rates of hospitalisation, admission to intensive care units (ICUs) and mortality compared to individuals without obesity in the recent COVID-19 pandemic [7].

The prevalence of overweight or obesity is alarmingly high, affecting 37% of the global population, with an even higher prevalence of 60% in high-income countries [8]. Indeed, as a country’s economic status increases, the national prevalence of overweight and obesity tends to rise significantly [8]. Furthermore, the global burden of obesity is rising, as evidenced by the increasing disability-adjusted life years (DALYs) and mortality rates, whilst malnutrition-related DALYs and mortality rates declined from 2000 to 2019 [9]. This trend is expected to continue, with the global number of CVD deaths attributable to high BMI projected to increase from 1.90 million in 2021 to 3.43 million in 2041, due to population growth, the increasing prevalence of obesity, and ageing populations [10].

Not only is this issue pervasive in adults, with 890 million adults reported as obese in 2022, but high BMI is also an issue among children and adolescents (aged 5–19) [3]. Globally, 390 million children and adolescents were overweight and 160 million were obese in 2022 [3]. Obesity is a critical public health concern, and despite advancements in medical care, it remains the fifth leading cause of death worldwide [6, 11, 12].

Obesity in Arab countries is a particular health concern, with prevalence estimated at 28% in 2016, a 10%-point increase from 2000 and double the global prevalence of 13% [13]. In a recent study, the Gulf Center for Disease Control and Prevention identified obesity as the leading public health priority among non-communicable disease risk factors [14]. Gulf Cooperation Council (GCC) countries report the highest rates of obesity globally, and in 2018, the Eastern Mediterranean Region (EMR) had the third-highest global prevalence of obesity [15, 16]. In eight countries of the EMR, obesity was the risk factor responsible for the highest number of DALYs in 2019, and the second highest for a further seven countries [9]. As well as health impact, the high prevalence of overweight and obesity across Arab countries is associated with considerable economic costs for both the individual and wider society [15].

The modernisation, urbanisation and rapid development of Arab countries have played a role in the recent rising prevalence of obesity [17]. For instance, westernisation and an increased influence from social media have led to a shift from traditional Arab diets to an increased consumption of processed, fast foods within the population [17, 18]. Modernisation has led to the devaluing of physical activity, with exertion associated with low-status occupations [17].

Whilst the interventions for obesity remain the same – dietary changes and physical activity complemented by therapeutic agents – contextual considerations must be made for the populations of Arab countries [12]. The desert climate challenges the feasibility of exercising outdoors, and whilst gymnasiums are becoming popular, cultural barriers, especially for women, restrict access to such facilities [17, 18].

Given this context, it is crucial to highlight the trends in high BMI-related mortality and disability within Arab countries. This study seeks to investigate the regional burden of high BMI-attributable disease and compare it with global data, to shed light on this public health issue and inform future policies.

## Methods

### Data sources

Data for this study were extracted from the Global Health Data Exchange GBD Results Tool (https://www.healthdata.org/research-analysis/gbd) [19]. This tool was developed to enable a comprehensive evaluation of age- and sex-specific mortality for 288 causes, prevalence, and years lived with disability for 371 diseases and injuries, as well as comparative risks for 88 risk factors across 204 countries and territories, and 811 subnational locations from 1990 to 2021 [20]. Detailed methodologies for the GBD 2021 and the comparative risk assessment, specifically for high BMI, have been described elsewhere [20, 21].

The GBD 2021 study used the protocol published on the Institute for Health Metrics and Evaluation website and described in similar literature studying BMI [19–22]. Additional analysis performed by the authors of this study involved applying GBD data to investigate high BMI-attributable disease and its impact in 22 Arab countries (Algeria, Bahrain, Comoros, Djibouti, Egypt, Iraq, Jordan, Kuwait, Lebanon, Libya, Mauritania, Morocco, Oman, Palestinian Territories, Qatar, Saudi Arabia, Somalia, Sudan, Syria, Tunisia, United Arab Emirates, and Yemen). All GBD 2021 analyses complied with the Guidelines for Accurate and Transparent Health Estimates Reporting statement.

### Definitions

The GBD 2021 study estimated the impact of high BMI by comparing actual health outcomes to hypothetical outcomes under historical exposure scenarios. This approach helped estimate the excess burden of disease attributable to rising BMI over time. BMI is calculated by dividing an individual’s weight in kilograms (kg) by the square of their standing height in metres (m), giving a value in kg/m^2^. A high BMI in the GBD 2021 study is defined as a value greater than or equal to 25 kg/m^2^ [20]. Within the GBD 2021 study, causes of high BMI attributable health burdens are classified as per a four-level hierarchy, detailed previously [22]. For this study, Level 2 categories of disease were analysed, for example, CVD or the composite of diabetes and kidney disease.

In this analysis, disease burden is assessed using three indicators: death rate (also referred to as mortality), DALYs and years of life lost from mortality (YLLs). Here, DALYs are reported as a rate per 100 000 population. However, they are typically understood as a cumulative measure of years lost to premature mortality and disability, i.e., less-than-optimal health. YLLs are described using the same rate but quantify the years of life lost to premature mortality. Alongside mortality rate, these provide a comprehensive analysis of disease burden through the impact on quality of life.

### Analysis

For both global data and that from Arab countries, age-standardised data were available for male and female sexes. Causes of mortality, DALYs and YLLs were presented as rates per 100 000 population, comparing changes over a 30-year period between 1990 and 2021. Global and Arab country data were compared.

### Role of the funding source

Not applicable, no funding for this study.

## Results

### Deaths attributable to high BMI

**Fig. 1:**
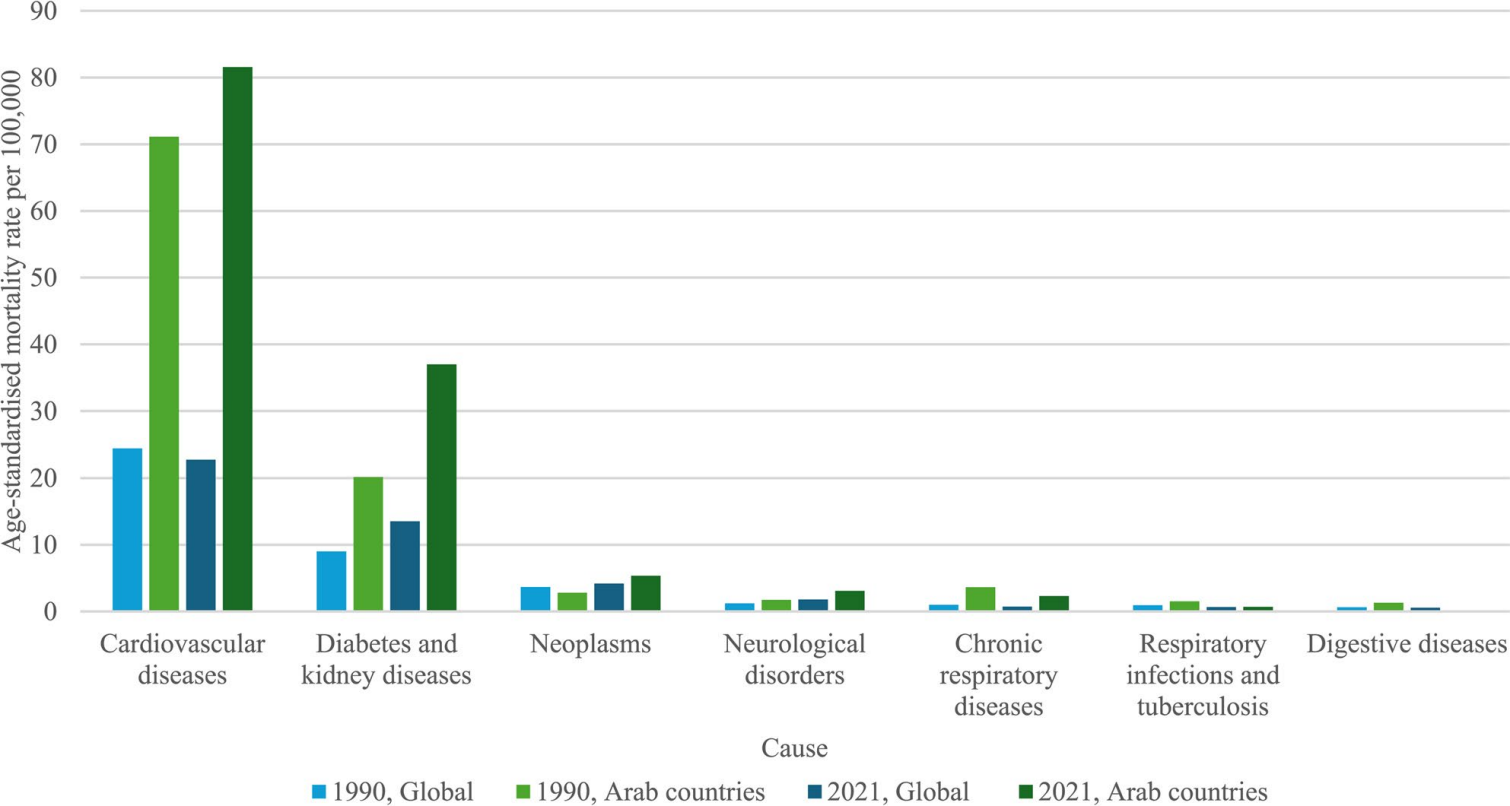
Age-standardised mortality rate per 100 000 population, by cause, both sexes

Global GBD data showed that the top three causes of age-standardised deaths attributable to high BMI, in both 1990 and 2021, were CVD, diabetes and kidney diseases and neoplasms (Fig. 1). In 1990, the mortality rates per 100 000 population were 24·43, 9·01, and 3·66, respectively, for these three causes of death. In 2021, mortality rates per 100 000 population were 22·77, 13·51, and 4·18, respectively. Other, less substantial, causes of high-BMI attributable deaths throughout this period include neurological disorders, chronic respiratory diseases (CRD), respiratory infections, tuberculosis and digestive diseases.

In Arab countries, in 1990, the top three causes of global age-standardised deaths attributable to high BMI were CVD, diabetes and kidney diseases, and CRD, while in 2021, neoplasms replaced CRD (Fig. 1). In 1990, the mortality rates were 71·14, 20·16 and 3·64 per 100 000 population for the top three causes of death. In 2021, the mortality rates per 100 000 population for the top three causes of death were 81·57, 37·00, and 5·35 respectively.

**Fig. 2:**
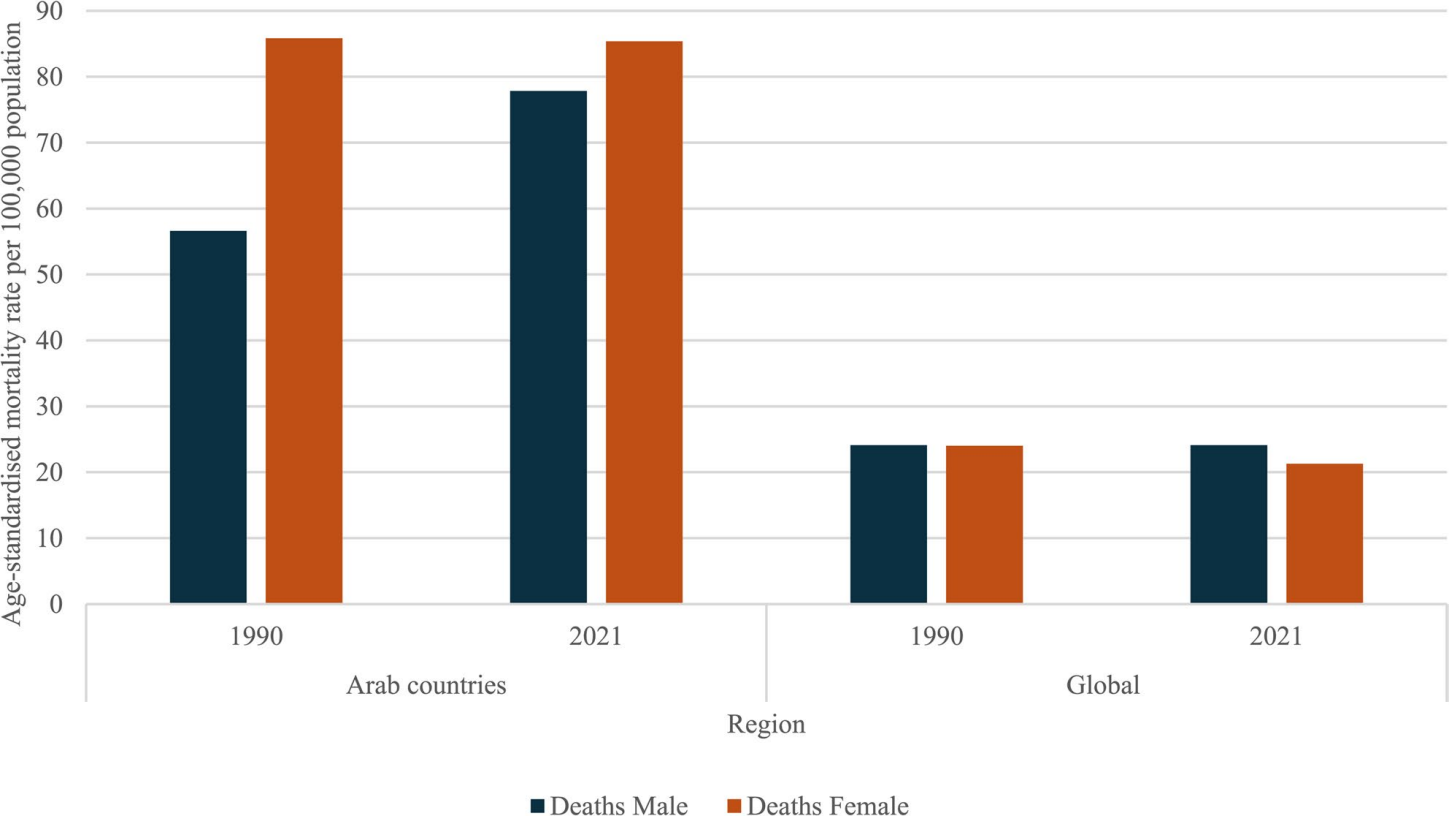
Age-standardised mortality rate per 100 000 population, for CVD, males and females

For each major cause, overall high-BMI attributable mortality across the two sexes was compared, both globally and in the 22 Arab countries. There was little difference between the sexes at a global level for CVD (Fig. 2), with mortality rates per 100,000 of 24·09 among males and 24·01 among females in 1990, and 24·09 among males and 21·29 among females in 2021. In contrast, in the Arab countries, mortality rates were 56·62 and 85·82 per 100 000 among males and females, respectively, in 1990. The 2021 rates were 77·83 and 85·38 among males and females, respectively.

**Fig. 3:**
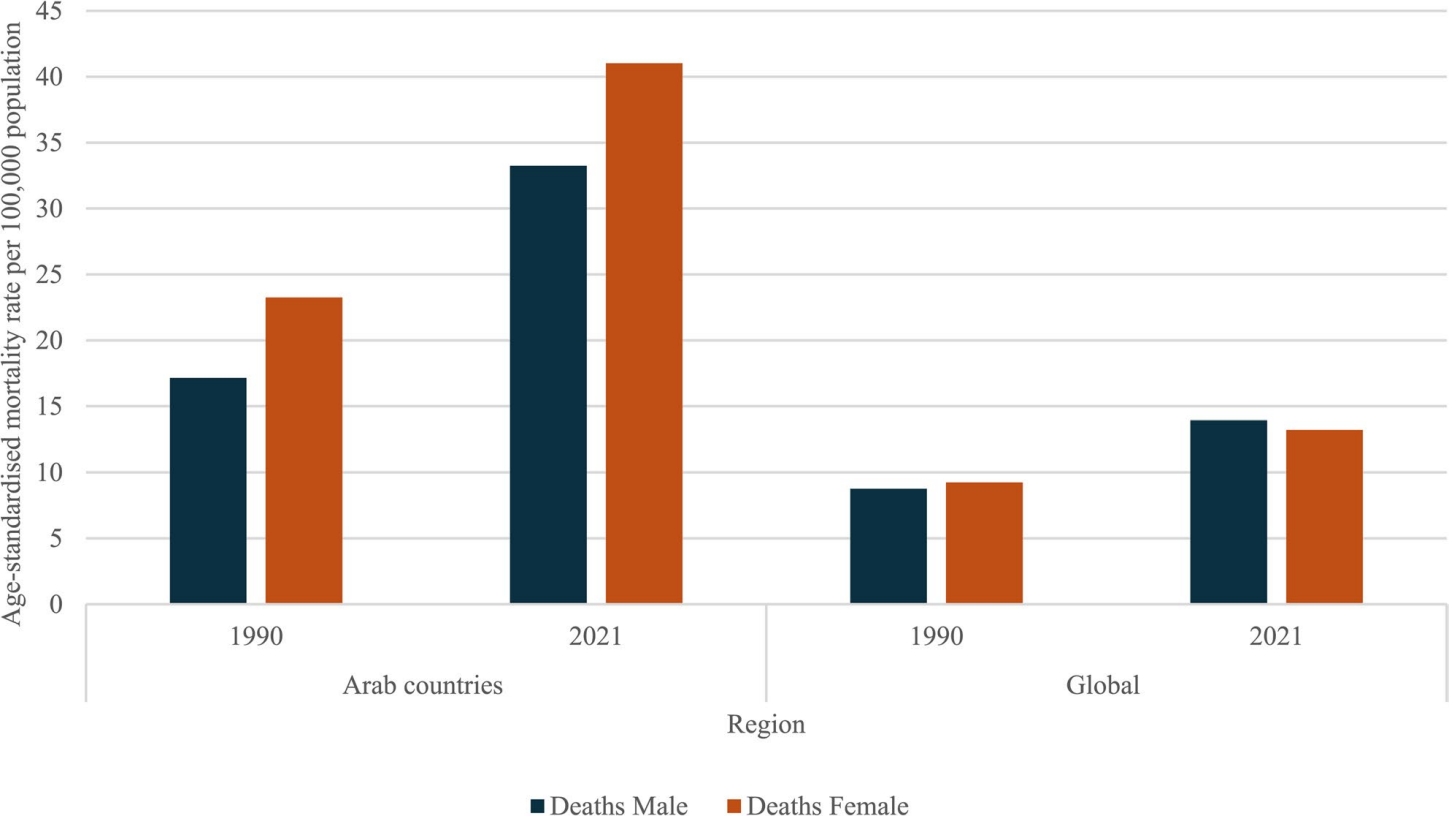
Age-standardised mortality rate per 100 000 population, for diabetes and kidney diseases, males and females

The differences by sex observed for diabetes and kidney diseases were comparable (Fig. 3). In 1990, the global mortality rates for males and females were 8·76 and 9·25 per 100,000, respectively, and by 2021, these rates had risen to 13.93 and 13.22, respectively. In Arab countries, mortality rates were 17·15 and 23·26 per 100 000 among males and females respectively in 1990, and in 2021 rates were 33·25 and 41·02 among males and females respectively.

### DALYs attributable to high BMI

**Fig. 4:**
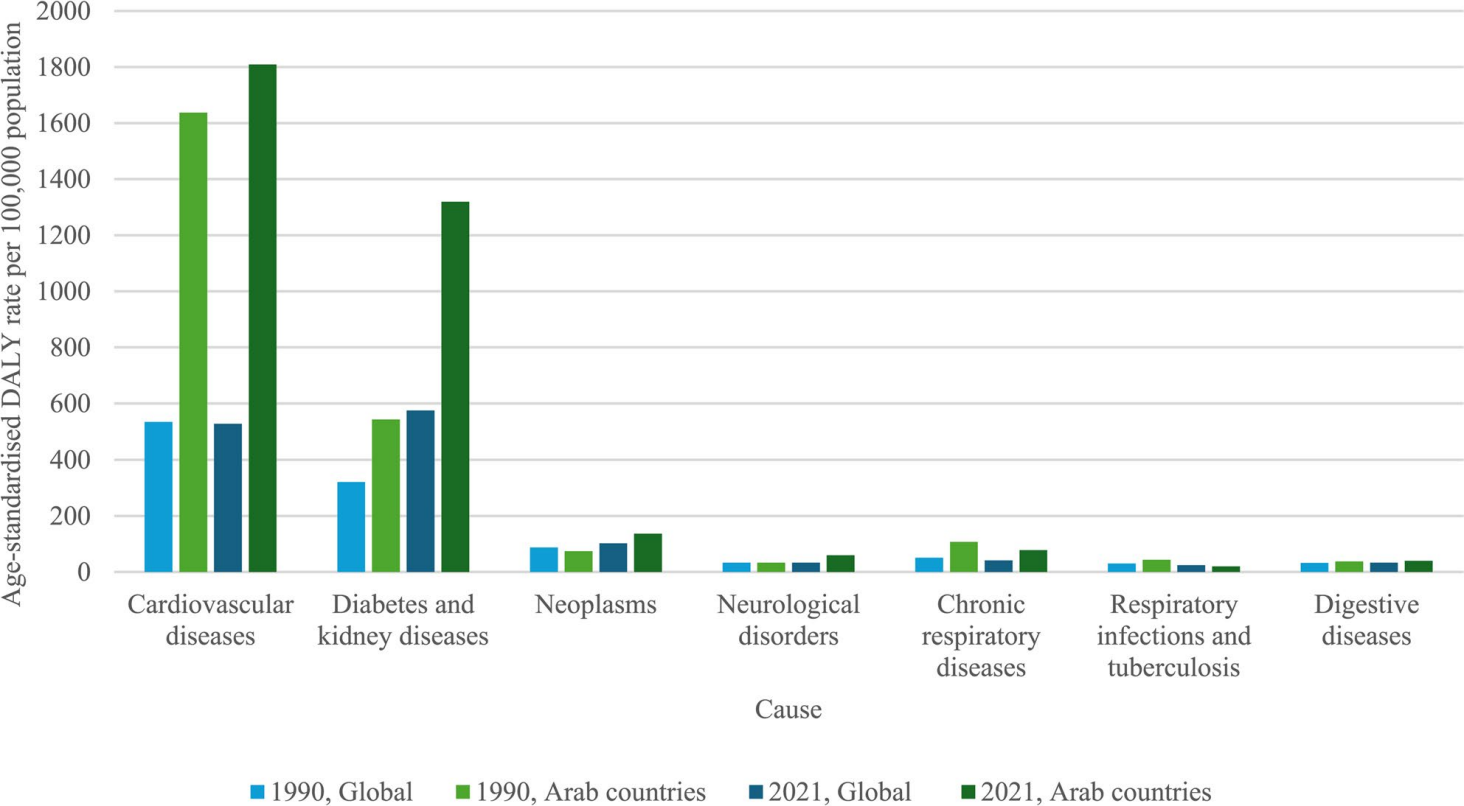
Age-standardised DALY rate per 100 000 population, by cause, both sexes

In 1990, the top three high-BMI attributable diseases causing DALYs globally were CVD, diabetes and kidney diseases, and neoplasms, with age-standardised DALYs per 100 000 of 535·01, 320·65 and 87·52, respectively (Fig. 4). In 2021, the same three diseases were the top causes of DALYs, with rates of 529·00, 574·62 and 102·16 per 100 000 population (Fig. 4).

In 1990, the top three high-BMI attributable diseases causing DALYs across the Arab countries were CVD, diabetes and kidney diseases, and CRD, with age-standardised DALYs per 100 000 of 1 636·96, 544·41 and 107·22, respectively (Fig. 4). In 2021, the main causes of DALYs were CVD, diabetes and kidney diseases, and neoplasms, with age-standardised DALY rates of 1 807·79, 1 319·70 and 136·71 per 100 000 population (Fig. 4).

Consistently, rates were compared across sexes for the major causes of DALYs. At a global level, DALY rates for CVD in 1990 were 561·58 among males and 500·11 among females, per 100 000, and 587·75 among males and 469·49 among females in 2021 (Supplementary Materials). In Arab countries, DALY rates were 699·78 and 917·90 per 100 000 among males and females respectively in 1990, and in 2021 rates were 1258·09 and 1138·40 among males and females respectively.

Global DALY rates for diabetes and kidney diseases in 1990 were 312·47 among males and 327·85 among females, and in 2021 these rates had risen to 588·90 and 561·63 respectively (Supplementary Materials). In Arab countries, DALY rates were 544·41 and 700·11 per 100 000 among males and females respectively in 1990, and in 2021 rates were 1237·30 and 1409·04 among males and females respectively.

### YLLs attributable to high BMI

**Fig. 5:**
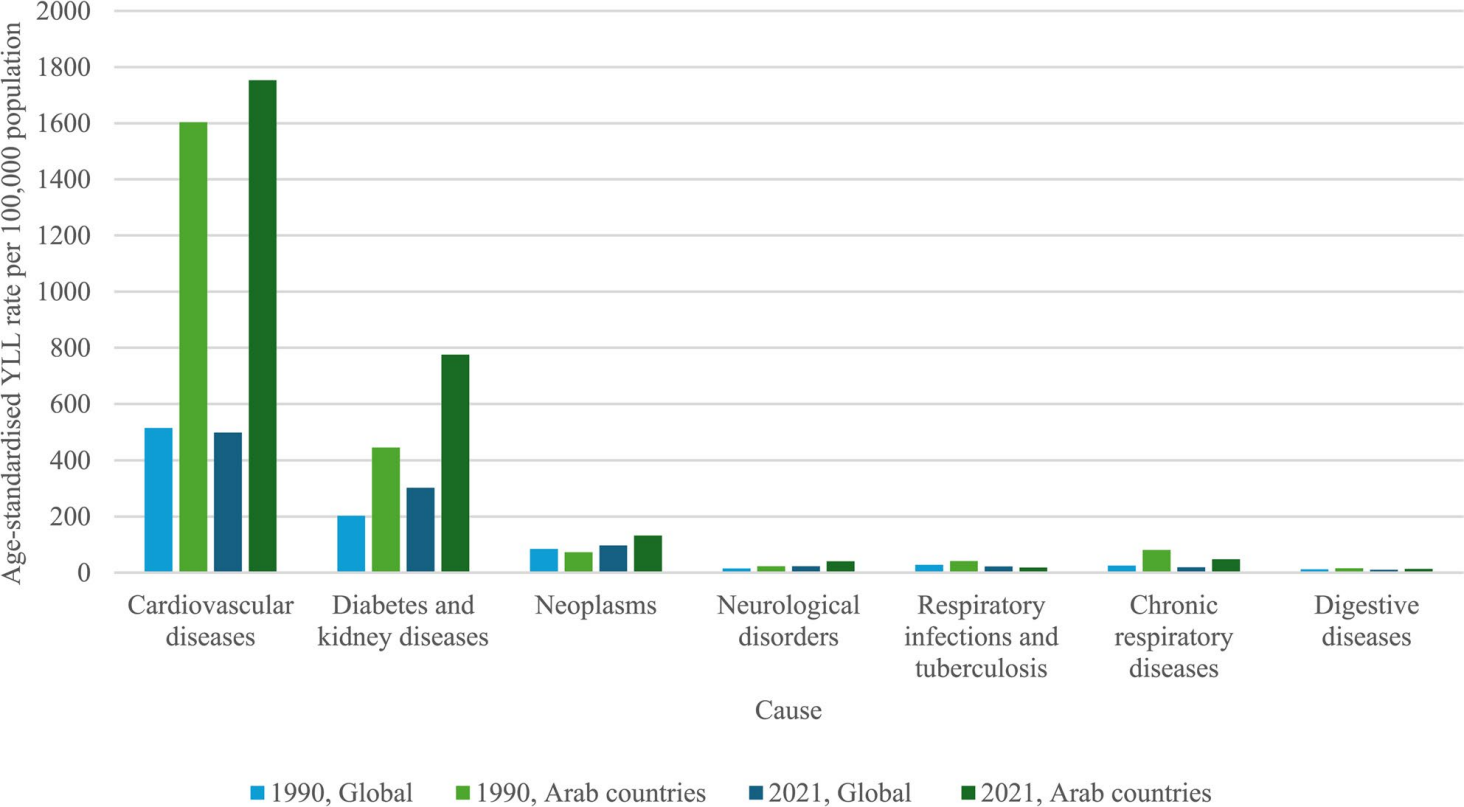
Age-standardised YLL rate per 100 000 population, by cause, both sexes

At a global level in 1990, the top three high-BMI attributable diseases contributing to YLL were CVD, diabetes and kidney diseases, and neoplasms, with age-standardised YLL per 100 000 of 515·36, 202·56 and 84·15, respectively (Fig. 5). In 2021, the same three diseases were the top causes of YLLs, with rates of 499·32, 302·18 and 97·34 per 100 000 population.

In 1990, the top three high-BMI attributable diseases contributing to YLL across Arab countries were CVD, diabetes and kidney diseases, and CRD, with age-standardised YLLs per 100 000 of 1603·31, 445·35 and 81·06, respectively. In 2021, the main causes of YLLs were CVD, diabetes and kidney diseases, and neoplasms, with age-standardised YLL rates of 1 753·33, 775·48 and 132·48 per 100 000 population.

Comparing CVD YLL rates across sexes at a global level demonstrates that the rates are consistently lower among females (478·90 per 100 000 population in 1990 compared to 543·81 among males, compared to 438·72 per 100 000 population in 2021 compared to 559·27 among males; Supplementary Materials). Among Arab countries, YLL rates across sexes have become more equal over time, largely due to an increase among males (from 1 357·46 per 100 000 in 1990 to 1 763·40 in 2021, compared to 1 856·06 and 1 734·89 among females).

Global YLL rates for diabetes and kidney diseases in 1990 were 196·19 among males and 208·33 among females, and in 2021 these rates had risen to 311·52 and 293·97 respectively (Supplementary Materials). In Arab countries, YLL rates were 384·62 and 508·49 per 100 000 among males and females respectively in 1990, and in 2021 rates were 708·66 and 847·41 among males and females respectively.

## Discussion

Our analysis shows that there are critical differences in the burden of disease attributable to high BMI when comparing the 22 Arab countries to global data. High BMI is a significant modifiable risk factor for numerous chronic diseases [5, 6], and its prevention and management can be effectively addressed through both individual-level interventions and comprehensive community-based health policies, as demonstrated by Finland’s multisectoral approach to obesity prevention [23]. In this 30-year analysis, CVD was the primary contributor to high BMI-attributable deaths. However, while the rate of CVD deaths attributable to high BMI decreased slightly at the global level over this period, the corresponding rate among Arab countries increased. Similarly, CVD was the primary contributor to high BMI-attributable age-standardised DALYs, both globally and across Arab countries. Finally, CVD was the main contributor to age-standardised YLLs globally and across Arab countries, but there was a slight drop in global rates, and an increase across Arab countries. Diabetes and kidney diseases were the second largest contributor to high BMI-attributable deaths, age-standardised DALYs and YLLs. Mortality rates attributable to diabetes and kidney diseases increased both globally and among Arab countries. While rates of age-standardised DALYs per 100 000 increased both globally and in Arab countries, the increase in the latter is particularly notable. Consistently, rates of age-standardised YLLs per 100 000 also witnessed a greater increase in Arab countries compared to globally.

Comparison across sexes revealed some important differences, with a higher rate of CVD deaths attributable to high BMI in females compared to males in Arab countries (85.38 versus 77.83 per 100 000 in 2021). This is much more pronounced disparity than the global data which showed a difference of only 0.08 per 100 000 between males and females. A similar phenomenon was noted with regard to the composite of diabetes and kidney diseases. Both sexes experienced an increase in death and DALY rates. However, both are higher in females compared to males in Arab countries (41.02 versus 33.25 per 100 000 mortality rates in 2021, compared to a global difference of 0.71 per 100,000 between males and females). These findings corroborate existing literature, which cite gender specific sociocultural factors as key contributors, including body image and cultural preferences, societal expectations, and obesogenic lifestyle shifts and urbanisation [24, 25].

The results were consistent with Bhagavayhula et al.,’s finding of high prevalence of CVD in the Middle East (ME), including Arab countries [26]. In fact, this area of the world has the youngest age group that is exposed to acute myocardial infarction (AMI), since the nine risk factors are higher in ME compared to globally [27]. Elevated apolipoprotein-B to apolipoprotein-A-I ratio is the principal risk factor, followed by smoking and high LDL (Low-Density Lipoprotein) to HDL (High-Density Lipoprotein) cholesterol ratio [27]. Other factors, including diabetes and hypertension, were more associated with AMI in women compared to men, which could explain the high disease burden among women globally and in Arab countries [27]. Furthermore, obesity, especially in the abdominal area, and depression were presented as additional risk factors for developing AMI among Arab people [27].

Increased prevalence of risk factors, such as a 43·3% prevalence of dyslipidemia, and an 18·7% prevalence of family history, were also identified in the ME [26]. A parallel increase in CVD morbidity and mortality and obesity was observed in GCC countries over 40 years [17]. This surge correlated with sociodemographic changes, a sedentary lifestyle, and a change in food eating habits [17]. Weather and climate change, particularly high temperatures in this region, play a significant role in the surge in obesity and the associated diseases. Extreme heat and climate change could contribute to obesity by reducing outdoor activities, leading individuals to remain indoors and adopting sedentary lifestyles. Climate change may also alter the food supply chain, by affecting various components, including agricultural output, food access, and food quality [28], thereby making healthy food less available and more expensive [29]. As a result, individuals shift toward ultra-processed foods that are cheap and profitable [29]. Other factors, such as buildings and city infrastructure, don’t allow sports activities; social gatherings over food, and parents who prefer educational activities over sports and physical activities for their children, also influence behaviours [17, 18].

Obesity prevalence is higher among women than men in Arab countries due to a combination of biological, sociocultural, and lifestyle factors. Women in the region often experience lower physical activity levels, influenced by cultural norms and limited access to exercise facilities [17, 18]. Additionally, dietary habits, including high-calorie traditional foods and lower nutritional awareness, contribute to weight gain [17, 18]. Hormonal differences also play a role, as oestrogen affects fat distribution and metabolism. Socioeconomic factors, such as education, employment opportunities, and healthcare access, further impact obesity rates among women [17, 18]. Multiple pregnancies make women more vulnerable to obesity-related diseases [18]. These findings align with previous research, which showed that prevalence of overweight and obesity is higher among women than men across both Africa and the EMR (regions in part included in Arab countries) and highlights the need for future public health strategies to consider the role of gender in high BMI and in the mediation of its effects [18, 20, 30].

Obesity is an important factor in the growing diabetes burden in the GCC region [30]. Estimated glomerular filtration rate (eGFR) and serum albumin-to-creatinine ratio (ACR) levels increase with the increasing BMI, suggesting obesity is a risk factor for development of chronic kidney disease (CKD) [31]. A meta-analysis found that obesity (but not overweight) was linked to lower eGFR (< 60 mL/min/1·73 m²) and increased albuminuria in adults with BMI greater than 30 kg/m² (and greater than 25 kg/m² in Asians) compared to those below these thresholds [32]. Additionally, each 1 kg/m² increase in BMI raised the risk of eGFR decline by 2% [32].

### Policy implications

While various policies have been introduced to address high BMI in Arab countries, their impact has been muted, potentially due to the predominance of “top-down” approaches [33]. Instead, emerging literature suggests that population-level prevention efforts that include multiple components and are “integrated at the policy level, the community level, and the interpersonal level” would be more successful [20, 33].

Restricting the choice of unhealthy foods and guiding the choice of healthier alternatives, through several complementary actions. Firstly, a tax levy on highly processed or sugary foods and drinks should be introduced [9, 15]. Secondly, the advertising of highly processed foods should be regulated. In addition, there should be regulation of retail food, including fast-food chains [17]. Conversely, a subsidy on fruit and vegetables as well as educational campaigns could increase the consumption of healthier food alternatives, particularly in children and adolescents. Front-of-pack labelling that simplifies nutritional information has been introduced to a limited extent in some Arab countries and could be expanded through broader legislation across the region [34].

In 10 out of 15 Arab countries, in excess of 40% of the population were found to be physically inactive, a prevalence of inactivity which is notably higher than other regions globally [35]. Several structural and environmental factors contribute to this trend, including a hot climate, inadequate public transportation, and a lack of pedestrian-friendly infrastructure, which all discourage outdoor physical activity [35]. Climate change will further exacerbate these conditions. Provision of adequate indoor facilities for adults, adolescents and children, including gender-segregated fitness facilities, are necessary to encourage participation; the presence of such facilities is particularly important in schools [35].

Studies suggest that sedentary lifestyles are favoured across many Arab nations, highlighting the need for broader societal change [17, 18, 35]. The growing sports industry across Arab countries presents an opportunity to promote public participation in sports. Furthermore, collaboration with faith and community and school-based interventions offer promise in tackling obesity among children and adolescents through physical activity as well as health and diet awareness-raising components [33, 35]. Involvement of celebrities, such as famous actors, singers, and influencers, in promotion of physical activity and healthy eating via social media may have an effect on children and adolescents’ lifestyles [36, 37]. More generally, low education is a key determinant of physical inactivity in Arab countries, suggesting that increasing educational attainment may have co-benefits related to BMI [17, 18].

In summary, a holistic approach that combines regulatory measures, infrastructure development and community engagement is needed to reduce the burden of high BMI-related disease and improve long-term public health outcomes.

### Limitations

General limitations of the GBD methodology are described in more detail elsewhere, one being the potential for data quality issues, particularly regarding countries with poorer health record-keeping [9, 20]. Specific limitations relating to this study include the use of BMI as a proxy for obesity, thus failing to account for differences in body composition, which have been noted across ethnic groups [20]. Self-reported height and weight data is used to calculate BMI, which can introduce social desirability or recall bias, however this has been mitigated against through use of a correction factor [20].

In addition, while it is helpful to analyse data at a regional level, Arab countries should not be interpreted as one homogenous area. Intra-regional variability, including socioeconomic and health system profiles, should be accounted for when designing public health strategies.

### Suggested future studies

Future studies should explore other determinants of BMI-attributable disease, including race, ethnicity and socioeconomic status, reflecting the diverse populations of Arab countries, especially with the suggestion that Arab individuals have a genetic predisposition to obesity [11]. Further regional research among children and adolescents would be valuable in monitoring this public health challenge, as demonstrated by The WHO European Childhood Obesity Surveillance Initiative (COSI), which tracks and reports overweight and obesity burden among children in European countries [38]. Qualitative research into perceptions and causes of high BMI within Arab countries would also shed light on further nuances and allow for further optimisation of future interventions. The impact of conflict and food insecurity in Arab countries would also be valuable to explore. Finally, longitudinal studies that evaluate both the short- and long-term impacts of regulatory, educational, and cultural interventions would provide a more comprehensive understanding of their effects and inform the shaping of future successful interventions.

## Conclusions

In conclusion, this study provides an in-depth analysis of the burden of disease attributable to high BMI across Arab countries, in the context of global data. The increasing regional rates of deaths, DALYs and YLLs attributable to high BMI warrant further monitoring and contextualised public health responses tailored to the region’s specific demographic, socioeconomic, and environmental challenges.

## Supplementary Information

Supplementary Material 1.

## Data Availability

GBD study 2021 data resources are available online from GBD interactive data visual tool ([https://vizhub.healthdata.org/gbd-results](https://vizhub.healthdata.org/gbd-results)). GBD study 2021 data resources are available online from GBD interactive data visual tool ([http://ghdx.healthdata.org/gbd-results-tool](http://ghdx.healthdata.org/gbd-results-tool)).

## Abbreviations

ACR: Albumin-to-Creatinine Ratio
AMI: Acute Myocardial Infarction
BMI: Body Mass Index
CDR: Chronic Respiratory Diseases
CKD: Chronic Kidney Disease
CVD: Cardiovascular Diseases
DALYs: Disability-Adjusted Life Years
eGFR: Estimated Glomerular Filtration Rate
EMR: Eastern Mediterranean Region
GBD: Global Burden of Disease
GCC: Gulf Cooperation Council
HDL: High-Density Lipoprotein
ICU: Intensive Care Units
LDL: Low-Density Lipoprotein
ME: Middle East
MI: Myocardial Infarction
NAFLD: Non-Alcoholic Fatty Liver Disease
NASH: Non-Alcoholic Steatohepatitis
OSA: Obstructive Sleep Apnoea
YLLs: Years Of Life Lost
