# Supplementary material for: The burden of disease attributable to high body mass index across Arab countries: an analysis of data from the global burden of disease study 2021
Source: BMC Public Health. 2025 Nov 26;25:4331. doi: 10.1186/s12889-025-25620-w (PMC12751261; doi:10.1186/s12889-025-25620-w)
Supplement: Supplementary file 1 — Supplementary Material 1. [file 12889_2025_25620_MOESM1_ESM.pdf]

The burden of disease attributable to high body mass index across Arab countries: an analysis of data from the Global Burden of Disease Study 2021

Supplementary material

This supplementary file includes graphs displaying the age-standardised DALY and YLL rates per 100 000 population for CVD and diabetes and kidney diseases, split by sex.

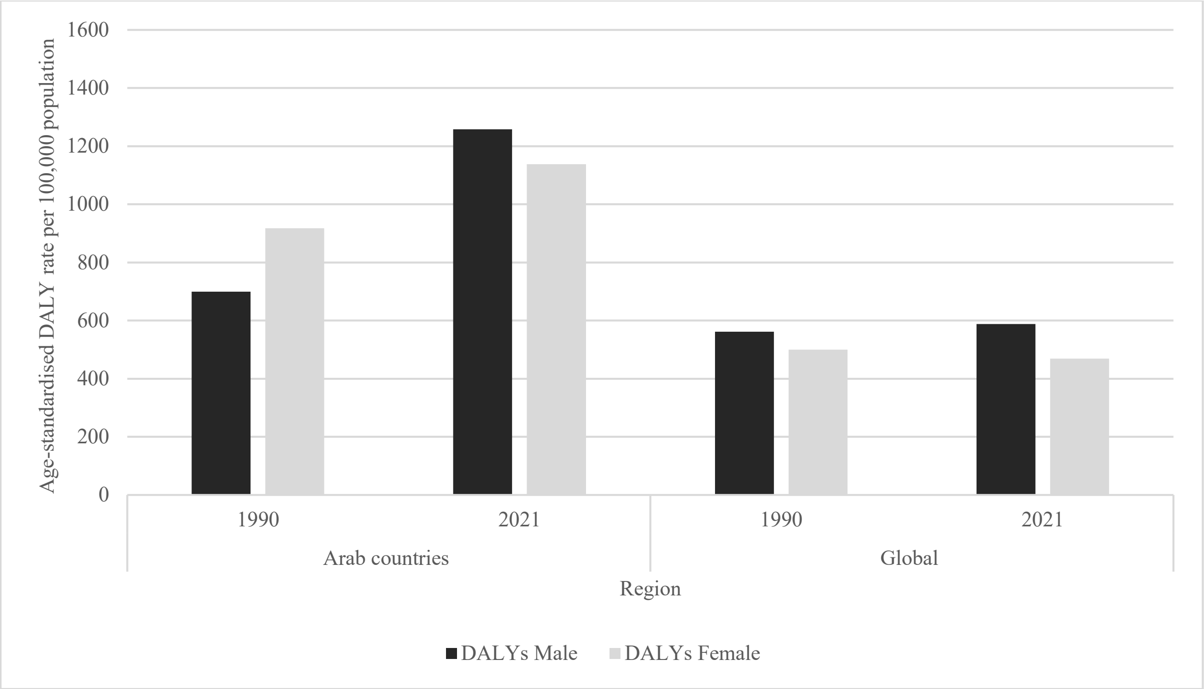

Figure S1: Age-standardised DALY rate per 100 000 population, for CVD, males and females

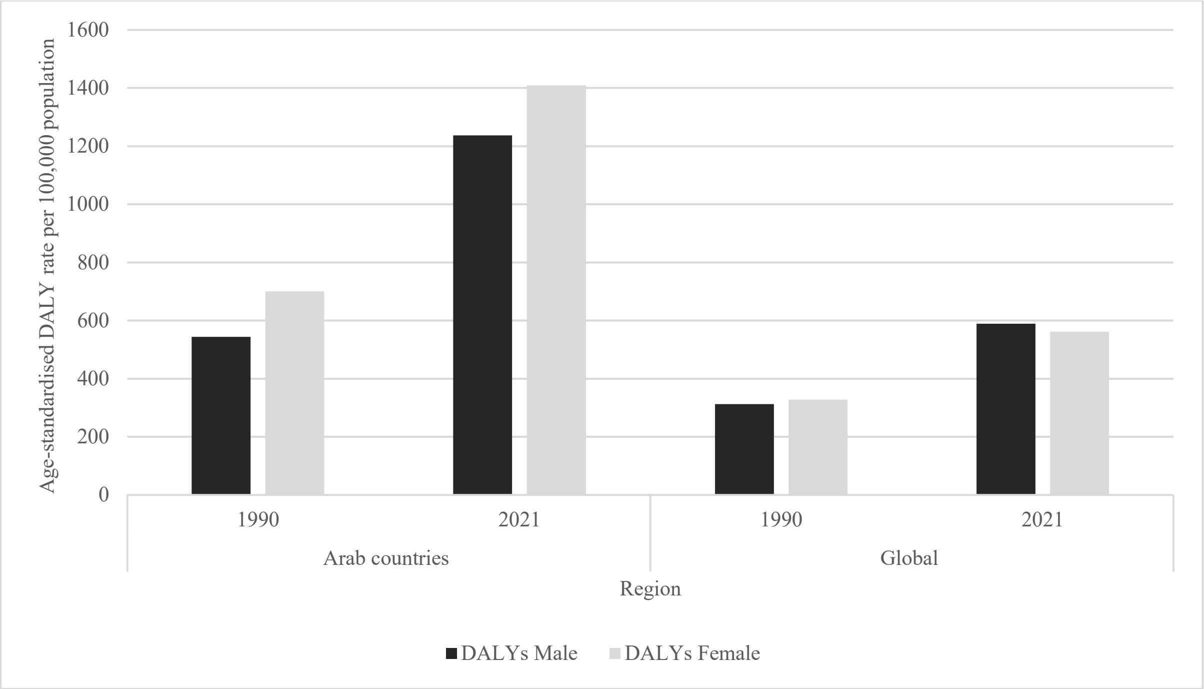

Figure S2: Age-standardised DALY rate per 100 000 population, for diabetes and kidney diseases, males and females

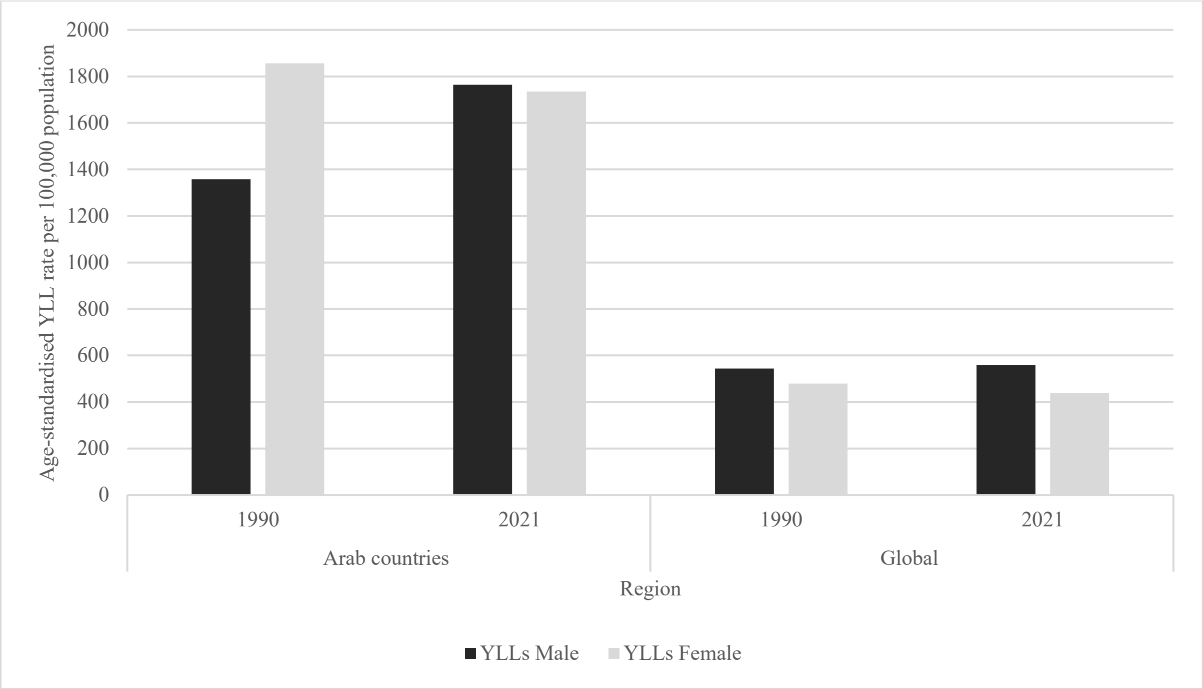

Figure S3: Age-standardised YLL rate per 100 000 population, for CVD, males and females

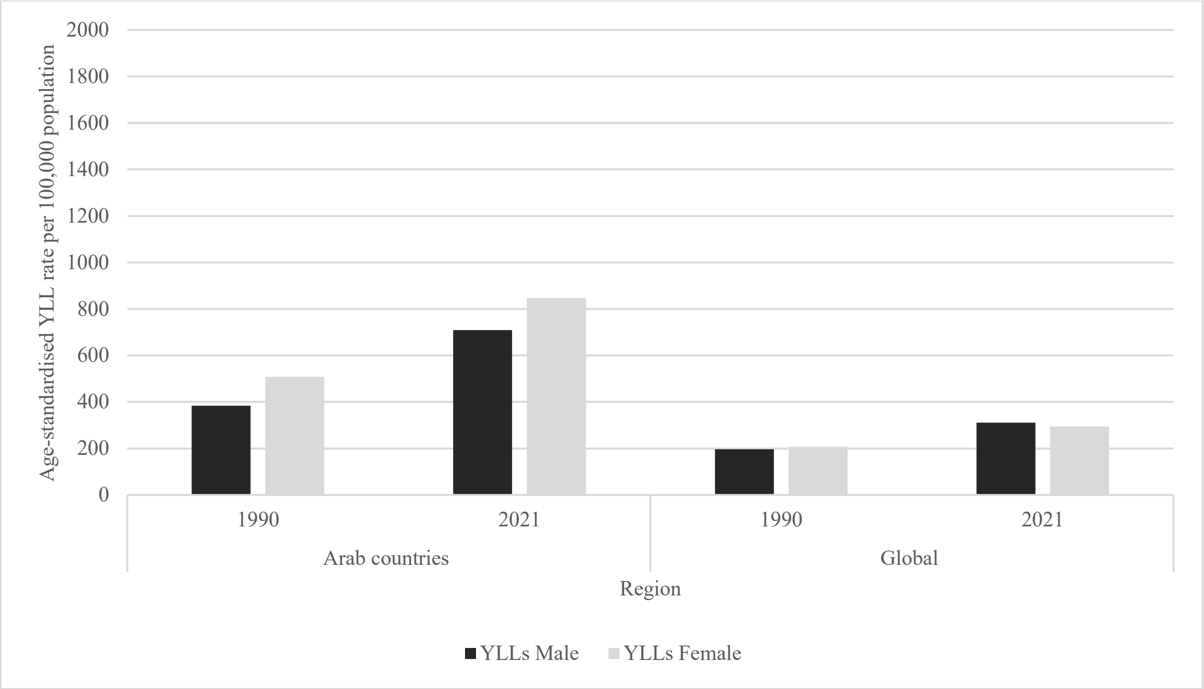

Figure S4: Age-standardised YLL rate per 100 000 population, for diabetes and kidney diseases, males and females
